# A Universal Testbed for IoT Wireless Technologies: Abstracting Latency, Error Rate and Stability from the IoT Protocol and Hardware Platform

**DOI:** 10.3390/s22114159

**Published:** 2022-05-30

**Authors:** Edgar Saavedra, Laura Mascaraque, Gonzalo Calderon, Guillermo del Campo, Asuncion Santamaria

**Affiliations:** CeDInt-UPM, Universidad Politécnica de Madrid, Campus de Montegancedo, Pozuelo de Alarcón, 28223 Madrid, Spain; lmascaraque@cedint.upm.es (L.M.); gcalderon@cedint.upm.es (G.C.); gcampo@cedint.upm.es (G.d.C.); asun.santamaria@upm.es (A.S.)

**Keywords:** testbed, latency, error rate, stability, performance, IoT, IIoT, LPWAN, wireless communications

## Abstract

IoT applications rely strongly on the performance of wireless communication networks. There is a wide variety of wireless IoT technologies and choosing one over another depends on the specific use case requirements—be they technical, implementation-related or functional factors. Among the technical factors, latency, error rate and stability are the main parameters that affect communication reliability. In this work, we present the design, development and validation of a Universal Testbed to experimentally measure these parameters, abstracting them from the wireless IoT technology protocols and hardware platforms. The Testbed setup, which is based on a Raspberry Pi 4, only requires the IoT device under test to have digital inputs. We evaluate the Testbed’s accuracy with a temporal characterisation—accumulated response delay—showing an error less than 290 µs, leading to a relative error around 3% for the latencies of most IoT wireless technologies, the latencies of which are usually on the order of tens of milliseconds. Finally, we validate the Testbed’s performance by comparing the latency, error and stability measurements with those expected for the most common IoT wireless technologies: 6LoWPAN, LoRaWAN, Sigfox, Zigbee, Wi-Fi, BLE and NB-IoT.

## 1. Introduction

In the last few years, the IoT has been established as one of the most acknowledged paradigms, increasing the amount of related research and emerging technologies and services [1,2], both regarding IoT devices —35 billion (×10^9^) devices connected in 2021 [3] and 75 billion devices expected by 2025 [4]—and monetary spending—more than EUR 1200 billion by 2027 [5].

The wireless paradigm for IoT allows medium and large coverage areas with relatively low energy consumption by providing small processing power requirements for devices as well as low transmission data rates [6,7], factors inherent in the IoT field itself. Despite the wide variety in wireless IoT technologies, some actors widely dominate the current picture of IoT communications, although this has always depended on the specific use case. Medium-range communications such as Bluetooth and Zigbee may have accounted for up to 28% of the wireless IoT chips in 2021 [8]. For long-range communications technologies, only four accounted for over 96% of global, installed active devices in 2021: NB-IoT, LoRa, LTE-M and Sigfox. NB-IoT leads this ranking with 47% of the global share, followed by LoRa with 36% [9]. In fact, low-power wide-area network (LPWAN) protocols that rely on licensed bands (NB-IoT, LTE-M) have surpassed those relying on non-licensed ones (LoRa, Sigfox) in 2021 [10].

However, different technologies provide different levels of performance and need different infrastructure requirements. Choosing one over another widely depends on the specific use case [11], and it is not always clear how to compare their performance. The very specific use case will set the requirements for wireless technology. Requirements may be divided into technical factors (data rate, latency, range), implementation factors (cost, documentation, available coverage) and functional factors (energy consumption, location services, over-the-air upgrade) [12,13,14,15]. Into the bargain, exceptional attention must be paid to security, especially considering the rapid growth of IoT and its more-than-ever quasi-omnipresent presence in our lives. The research presented by Anand et al. in [16] and that presented by Malhotra et al. in [17] are great references regarding the security challenges in the IoT field.

Testbeds allow us to determine the realistic behaviour of IoT systems and even foresee future possible upgrades and enhancements to the systems. As IoT systems are intrinsically wide in nature, so are their possible characterisation targets, which make it arduously hard to develop and validate universal performance tests for IoT in different matters.

In the literature, one can find testbed systems evaluating some specific IoT characteristics, usually focused only on a few IoT technologies, for instance, the work of Pereira et al. [18], in which an experimental characterisation of mobile IoT latency is carried out, or that by Mroue et al. [19], evaluating LoRa, Sigfox and NB-IoT in a MAC layer-based approach.

There are also deep characterisations of specific IoT technologies for a relatively wide range of matters, such as the survey by Rashmi Sharan et al. of LoRa and NB-IoT [20]; or the work carried out by Alsukayti et al. [21], in which they analyse quality, transmission range, power consumption and data rates for different scenarios and technologies. On top of that, there are works evaluating different features, and challenges to face with eventual low-latency or high-reliability IoT communication networks, such as those in [22,23,24].

Yet, there is not sufficient research in the literature about actual, universal, ubiquitous, accountable testbeds. This may be due, indeed, to the massively wide nature of the IoT field. Hossain et al. propose a manner to overcome this issue with the work presented in [25], in which a large-scale IoT testbed-as-a-service is defined. Developing testbeds that can integrate various types of systems, interfaces and technologies is tough but still needed for a field with more variety and presence every day. This fact is clearly highlighted in [26], where the authors emphasise the lack of interoperability among IoT platforms and devices. 

Specifically, no universal testbed regarding temporal end-to-end characterisation for IoT wireless technologies can be found in the literature. With this work, we want to abstract the IoT wireless technology characteristics as much as possible, providing a straightforward way of comparing wireless IoT technologies in different communication features: latency, error rate and stability. We developed a universal Testbed based on a Raspberry Pi 4 (RPi), which only requires the IoT device under test to have digital inputs (GPIO)—fact that can be taken for granted for virtually every IoT node. In the scope of this work, we analysed the performance of the following IoT wireless technologies: 6LoWPAN, LoRaWAN, Sigfox, Zigbee, Wi-Fi, BLE and NB-IoT.

The rest of the paper is organised as follows: Section 2 describes the Testbed and the workaround for this paper. In Section 3, we characterise the performance of the Testbed to determine its temporal accuracy and precision, i.e., error range. Section 4 presents real measurements and results from our Testbed for the wireless technologies under consideration. Finally, in Section 5, we briefly conclude the results of this work and discuss future milestones.

## 2. Universal Testbed

In this Section, the Testbed environment is described, both regarding the Testbed device itself and the laboratory set. First, we explain the IoT devices and configurations deployed for the different wireless technologies, and how *latency* must be considered on a user-level approach (Section 2.1). Then, we tackle some caveats regarding the wide variety of wireless technologies to consider (Section 2.2). Finally, in Section 2.3, the Testbed’s user interface and measurement data presentation are explained.

### 2.1. Latency and Laboratory Workaround

**Figure 1:**
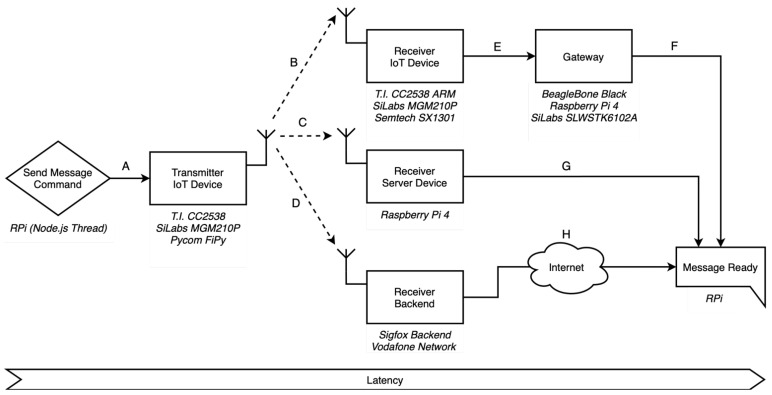
*Latency*: abstract definition and message paths for different wireless IoT topologies. These paths, along with the designated physical IoT devices—which are all compliant with the IoT technology of interest—actually portray the very use case deployed in the lab to do this work. Other configurations could be also used for certain technologies and other devices as well.

Since distinct wireless IoT technologies have different topologies and key network components, we first need to define *latency*. Pure upload messages are considered as they are the main purpose of the majority of IoT applications: sending information from the node to the user-end. So, for this scope, let us define *latency*—see Figure 1.

**Latency:**  *The time a message takes from the moment when the transmitting device is called to send the message until the message is ready for utilisation at the other end (user-side).*

**Figure A1:**
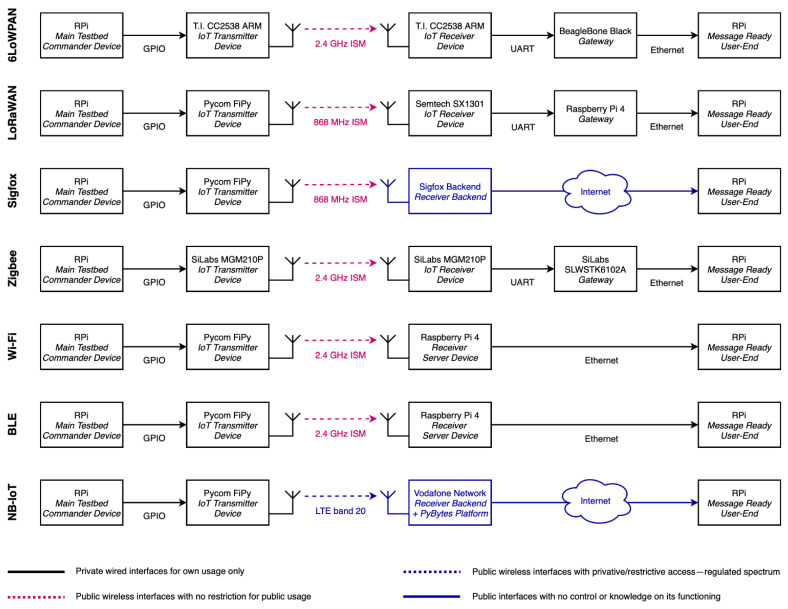
Detailed message paths for the wireless technologies characterised in this work, depicting the devices and interfaces used in each step. Note that other configurations—provided they comply with the standard—could be used, but these are the ones actually deployed in the laboratory for characterisation and Testbed validation. We also wanted to implement the most local approach possible in order to have more control over the message path.

Hence, time considers neither the acknowledgement (ACK) of the message nor possible retransmissions. Provided the message were lost, the transmission would count as an error. In this manner, considering the different topologies regarding different wireless technology’ specifications, three main groups can be defined: (1) technologies requiring a specific gateway; (2) technologies requiring public Internet usage; and (3) technologies requiring neither. Therefore, these groups (see Figure A1 for a detailed version on each technology) correspond to one of the following paths:

1. **A-B-E-F**: 6LoWPAN, ZigBee, LoRaWAN
2. **A-D-H**: Sigfox, NB-IoT
3. **A-C-G**: Wi-Fi, BLE

Notice that we did not use any routing protocol or routing device so as to make all wireless technologies point-to-point from the transmitter device to the receiver one. In Table 1, we can see the physical devices used for every wireless technology. The message commander is always the main RPi on which our Testbed software runs.

The first step from the RPi to the transmitter device is a wired link using two GPIOs. We described a naïve protocol for this communication based on 2 one-way digital signals: *R* (for reset) and *S* (for sequence). The *S* signal fires a rising-edge interrupt on the IoT transmitter device, which increases the message sequence number and sends it if *R* is high or otherwise resets it for a new test.

The next steps (B, C, D) are the RF link and correspond to the communication between the IoT end-device and its receiver—what we can consider the *pure* IoT communication phase. This path can be between two *similar* devices (path B: 6LoWPAN, Zigbee, LoRaWAN; or path C: Wi-Fi, BLE) or between the end-device and a public *base-station-type* receiver device (path D: Sigfox, NB-IoT).

In the last steps, the message travels (F, G, H) back to the main RPi, which parses the data received for latency, error and stability calculations. All communications are local, except for the last piece in the case of Sigfox and NB-IoT (H), as their own nature and working principles imply the usage of the carrier’s infrastructure and the public Internet to finally return back to the RPi.

It is worth noting that there might be other possible topologies and configurations for the wireless technologies, especially for pieces E, F, G, H, as long as they comply with their specifications and requirements. This whole work is focused on the very setup we used for each wireless technology, bearing in mind our aim to make the message path as *local* and simple as possible in order to have the most control over it.

#### The Transmitter IoT Device’s Firmware

The firmware of the transmitter IoT device is straightforward: it only needs to listen to a rising-edge interrupt and send a message accordingly, taking into account the *S-R* signals protocol. As a reference, the following piece of code depicts an implementation of the required firmware in MicroPython used with FiPy devices (LoRaWAN, Sigfox, Wi-Fi, BLE, NB-IoT):

import …

S = Pin(‘P21’, mode = Pin.IN, pull=Pin.PULL_DOWN)

R = Pin(‘P22’, mode = Pin.IN, pull=Pin.PULL_UP)n = 0x0

def pin_handler(arg):

global n

n = 0x0 if R.value() == 0 else n+1

*send_message(n)*

return

try:

S.callback(Pin.IRQ_RISING, pin_handler)

except KeyboardInterrupt:

sys.exit(0)

Depending on the technology under characterisation, *send_message(n)* would call the required methods. As we are using point-to-point communications between the transmitter device and the receiver device, the message they exchange only contains a text string with the message sequence number as payload—the other parameters shown in Section 2.3.4 herein are set by the receiver device/gateway/backend to be delivered to the main RPi. In fact, the only task the *receiver* is in charge of is completing that information and steering it back to the RPi.

### 2.2. Considerations Regarding Different Wireless Technologies

Since different technologies behave differently, have different requirements and provide different support on the development board’s software, some aspects of implementation must be known by the reader:

- Sigfox: The message payload was always set to be 12 bytes—the maximum allowed;
- LoRaWAN: is the most common configuration of Spreading Factor (SF7), and bandwidth (125 kHz) was used;
- BLE: We used advertisements to spread the message (two transmissions for each message). We did this because several problems were encountered in timing, and too many losses appeared when advertising only once—too many even to consider BLE an IoT technology. However, we attribute this to the fact of not being able to use point-to-point messages and only advertisements, and advertisements were the only BL- supported feature by Pycom at the time of doing this work;
- NB-IoT: We used Vodafone SIM card, i.e., Vodafone LTE network, with the layer of Pycom Pybytes as a backend for receiving messages;
- Wi-Fi: Hypertext transfer protocol (HTTP) was used as the application layer to send messages as it is an utterly common layer in Wi-Fi utilisation;
- 6LoWPAN: User datagram protocol (UPD) was used as the top layer to send messages;
- Zigbee: The clean Zigbee stack was used.

### 2.3. The Testbed’s Interface

The Testbed’s user interface is based on representational state transfer (REST) requests. Therefore, our Testbed provides an HTTP endpoint on port 8080 to listen to REST requests—get, put, post. A Node.js instance—the Testbed’s interface backend—is listening to those requests so as to summon distinct actions accordingly. 

So far, the basic, essential functions have been implemented, aiming to develop a more user-friendly interface with deeper functionality in the near future. It is noteworthy that two main types of resources can be categorised: (1) those used by the users themselves, i.e., the pure user interface described in Section 2.3.1, Section 2.3.2 and Section 2.3.3 and (2) the resource needed by IoT devices to send messages and thereby measure latency, error and stability, as described in Section 2.3.4

#### 2.3.1. Start Test (Route: POST /start/{*idtech*}/period/{*period*}/limit/{*limit*})

This functionality must be invoked to start a new test for any wireless technology. The following parameters must be specified in the uniform resource identifier (URI) contents: wireless technology, period of transmissions and message limit, where:

- *idtech* is an integer designating the wireless IoT technology under test (required);
- *period* is the period between transmissions in milliseconds (defaults to 10,000);
- *limit* is the message limit (defaults to 100).

For instance, to start a 6LoWPAN test with half a second between messages and one thousand thereof, the request would be: /start/1/period/500/limit/1000.

#### 2.3.2. Print Current Results (Route: GET /print)

This functionality prints the test results thus far on a remote secure shell (SSH) terminal, with the test still running. The user must have established an SSH connection with the RPi to see the results. Although it might be counterintuitive, results are not part of the request’s response but a log in the SSH terminal. This functionality might be modified, as it seems more useful to send results alongside the REST response. They are presented in the same format as described in Section 2.3.3 herein. This resource is useful to check the proper performance of an ongoing test.

#### 2.3.3. Stop Test (Route: PUT/stop)

This invocation halts the current test, calculating the results and storing them in JavaScript Object Notation (JSON) format. The user may invoke this at any time to stop the ongoing test. The same functionality is auto-invoked if the test achieves the message limit, or any kind of exception occurs.

The JSON results file contains time (all timestamps in milliseconds) and loss information for every message as well as a summary with the most important information about the test. For instance, for the test in Section 2.3.1: 

{

“0”: {

“start”:         1601290079097.324,

“successful”:    true,

“end”:                1601290079127.223,

“timestamp”:     1601290079114,

“latency”:            29.899

},

…

“999”: {

“start”:        1601290579130.133,

“successful”:   true,

“end”:               1601290579152.390,

“timestamp”:    1601290579147,

“latency”:           22.257

},

“testInfo”: {

“testStart”:    1601290079071,

“testEnd”:         1601290579154,

“limit”:        1000,

“top”:               999,

“period”:            500,

“idtech”:            1,

“avgLatency”:    22.053,

“minLatency”:    19.573,

“maxLatency”:    283.493,

“stdDev”:        11.220,

“outliers”:            32,

“outliersRel”:    3.20,

“erorrCount”:     0

“errorRate”:      0.00,

“finishReason”:        “limit”

}

}

For this kind of file, there are two main data groups:

- Individual message data (fields identified by a numeral, from “0” to “999” in this example):
  - *start* is the timestamp when the IoT transmitter device was called to send the message;
  - *successful* is a Boolean indicating if the message properly arrived at its destination;
  - *end* is the timestamp when the RPi received the call-back from the IoT receiver device—the moment when the latency trip is completed;
  - *timestamp* is a timestamp recorded by some gateways when processing the messages (only for 6LoWPAN and LoRaWAN);
  - *latency* is the difference between *end* and *start*, i.e., the *latency* of the message.
- Whole test data (field “testInfo”):
  - *testStart* is the timestamp of the moment when the test began;
  - *testEnd* is the timestamp of the moment when the test finished;
  - *limit* is an integer indicating the number of messages defined in the test;
  - *top* is the maximum sequence number achieved when performing the test—it should be *limit*–1 if the test goes well;
  - *period* is the period between messages defined in the test configuration;
  - *idtech* is an integer designating the wireless technology;
  - *avgLatency* is the average *latency* for the whole test;
  - *minLatency* is the minimum registered *latency;*
  - *maxLatency* is the maximum registered *latency;*
  - *stdDev* is the standard deviation for the cluster of *latencies* recorded in the test;
  - *outliers* is the count of *latencies* out of *avgLatency* ±10%;
  - *outliersRel* is the relative number of outliers with respect to *top*+1;
  - *errorCount* is the messages lost count during the test;
  - *errorRate* is the relative error rate—*errorCount* with respect to *top*+1;
  - *finishReason* is the reason the test finished; usually, the test finishes because the message count (*top*) reaches its *limit.*

#### 2.3.4. Callback (Route: POST /callback)

This is the endpoint to which the device under characterisation (user-end) must steer its responses, also in JSON format. As stated before, this endpoint is located on the main Testbed device (RPi) to reduce temporal incongruences. 

These messages are used to calculate latency and losses—therefore error and stability as well. Response messages were designed to be very small—so as to not be affected by some wireless technologies’ highly restricted bitrates—and have the following format:

{

“id”: *idtech*,

“sq”: *seq*,

“ts”: *epoch_ms*

}

where:

- *idtech* is an integer designating the wireless technology;
- *seq* is the message sequence number, useful to sort messages upon arrival, measure latency and determine losses;
- *epoch_ms* is the timestamp of the device under characterisation in milliseconds—however, this time is not used to calculate this kind of *latency* as the Testbed device uses its own time reference for that.

## 3. The Testbed’s Temporal Characterisation

In this Section we analyse the temporal performance of our Testbed device, as timekeeping and congruence is crucial when characterising latencies in the order of a few milliseconds. In the upcoming Section 3.1, Section 3.2 and Section 3.3, the device is characterised precisely to demonstrate its ability to work with IoT typical latencies, determining its error range in Section 3.4.

According to the IoT wireless technology spec sheets and the previous literature [11,12,27], the fastest latencies to consider would be on the order of tens of milliseconds (~10 ms). Hence, with a conservative approach of *one order of magnitude* precision, the error of our Testbed device should be on the order of milliseconds (~1 ms) to properly be used in this scenario.

The main software of our Testbed is a Node.js application running on a Debian-based operating system (OS), which manages the requests and accordingly calculates the results for a given test. Neither Node.js nor Debian are real-time intended. That being so, they introduce a delay when reacting to events and performing actions. Furthermore, since Node.js is JavaScript-based, it is synchronous—special care with time-related matters must be taken [28].

To characterise the response time and stability of our Testbed, we evaluated three matters which are considered fundamental to properly portray the Testbed’s temporal response [29,30,31]:

1. Stability responding to external interrupts (Section 3.1);
2. Temporal stability of the internal clock (Section 3.2);
3. Auto-delay when responding to self-events (Section 3.3).

Our system introduces a certain delay every time it needs to react to some event or perform some action. This delay must be characterised accordingly to evaluate its influence on *latency* measurements. From these three parameters, we can evaluate the Testbed’s precision and accuracy.

### 3.1. The Testbed’s Reaction to External Interrupts

We used a waveform generator (Keysight 33220A) to make a 50 Hz square wave, with edges to trigger a GPIO interrupt (both rising and falling edge) on the RPi, meaning that the interrupt triggers 100 times a second—a 10 ms interrupt period. This frequency of events is far beyond what is needed for wireless IoT characterisation—one per second as much.

With this procedure, the RPi stores a timestamp in microseconds: this allows us to determine the temporal stability of the Testbed as we are using fixed-frequency square wave edges to trigger the interrupts.

We let the application run, reacting to 1.15 million interrupts and storing their microseconds timestamps; it ran for 192 min. Let us claim one million values are enough for characterising the stability of the system. From this array of interruption timestamps, we extracted the periods of trigger—the difference between every pair of timestamps.

Then, we analysed the data using Matlab. The array of periods was converted to a cluster of errors between the observed period by the RPi vs. the deterministic period of the waveform generator. The latter was double-checked using an oscilloscope (Keysight Infiniium MSO8064A), sampling at 1 MS/s with 256 averages. The oscilloscope read out a low-value width of 9.9555 ms and a high-value width of 10.0448 ms. 

**Figure 2:**
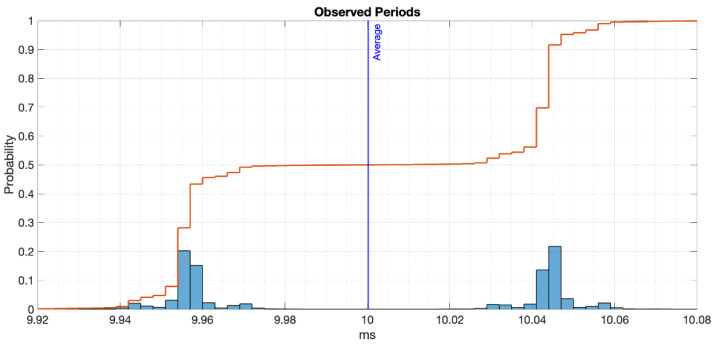
Histogram of the observed periods by the RPi reacting to an external 50 Hz square wave.

These values must correspond to the periods measured by the RPi. In fact, there are two main groups of observed periods when plotting the recorded data (see Figure 2), for which the cumulative distribution probability is half for each set, as expected with a 50%-duty-cycle square wave.

**Figure 3:**
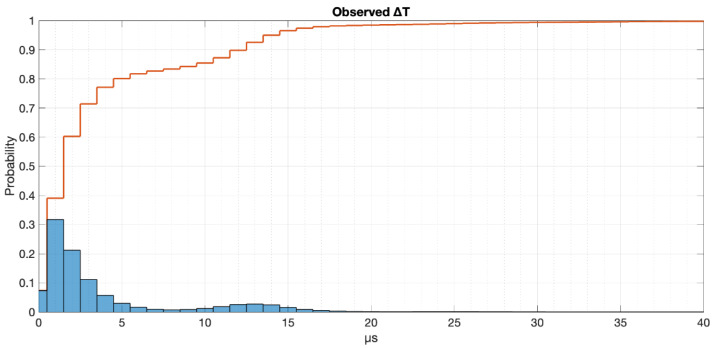
Histogram of the time drifting for both sides of the square wave.

Converting these values to differences of time with respect to the deterministic values of both the low part and high part of the square signal let us plot an error image of the observed periods. In Figure 3, we depict this fact assuming the average central value for splitting the set of values as the deterministic period. The absolute values of the time drift for both sides of the wave are plotted out as a whole (blue bars) as well as the cumulative probability (red line).

As one can see, the time drifting realised when reacting to external interrupts is only a few microseconds, being <5 µs for 80% of the times, <12 µs for 90 % of the times and <15 µs for 95 % of the times. Table 2 depicts the most important data for this characterisation.

### 3.2. Temporal Stability of the Internal Clock

With this characterisation, we want to evaluate the long-term temporal stability of the Testbed’s internal clock. We commanded the RPi to generate a 100 Hz square wave, toggling the logical value of a GPIO every 10 ms. 

This GPIO was connected to a Nordic Semiconductor Power Profiler Kit II (PPK) with a 3.3 kΩ series resistor to measure current—the device we already checked as valid laboratory equipment in [11]. We set the PPK to sample the signal at a rate of 10 kS/s, complying with the Nyquist–Shannon sampling theorem for signals up to 5 kHz.

The data gathered with the PPK was processed in Matlab to determine its fast Fourier transform (FFT). Since the recorded signal it is a square wave, it will have *infinite* harmonics, but the first one represents the fundamental frequency of the signal generated by the RPi, which is ideally 50 Hz—10 ms high, 10 ms low: 20 ms period.

**Figure 4:**
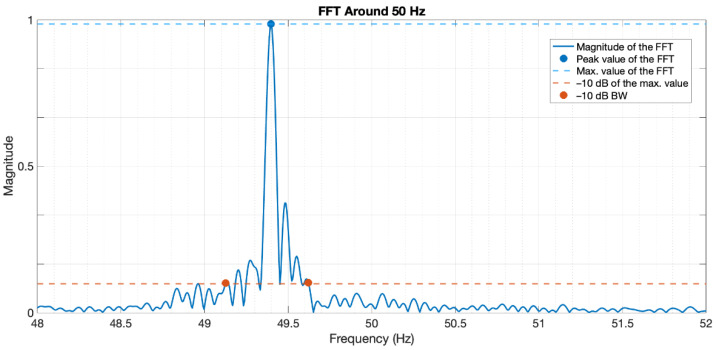
50 Hz centred FFT of the RPi-generated square wave.

In Figure 4, the FFT of the square wave generated by the RPi is depicted for a range of frequencies around the point of interest.

We can see that there is not only one peak at the desired frequency (50 Hz) but a predominant peak around 49.4 Hz with some small lobes around it—certain bandwidth (BW). This is due to the fact of the RPi not being 100% time-consistent. Moreover, the code itself and the OS resources spend some time that cannot be directly controlled. The magnitude of the signal does not show any units as the important matter is the relation in magnitude between the central frequency and the side lobes, and those values are also dependant on the FFT size. We also performed tests for other square wave frequencies between 10 Hz and 100 Hz, and the temporal deviation (in units of time) was coherent, which is the matter of interest for this analysis—not so, the frequency deviation, as it is relative to the frequency of the wave. Important data from this plot are shown in Table 3.

With this, the error related to the internal clock’s deviation can be claimed to have an average value of 240 µs, with 90% confidence values between 160 µs and 370 µs.

### 3.3. Self-Delay

**Figure 5:**
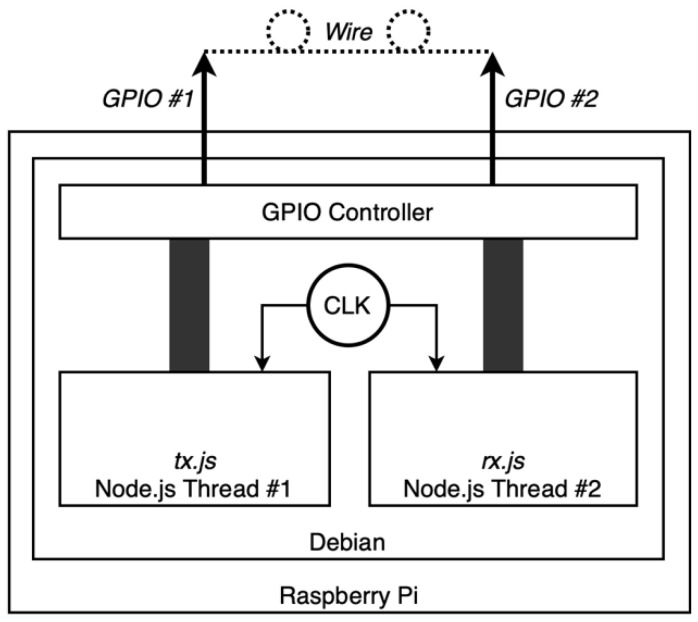
Hardware loop representation.

To characterise the delay of the proper master device itself, having no other external reference per se, we used something we called a *hardware loop*—see Figure 5.

The *hardware loop* connects two GPIOs from the RPi in a manner that the proper Testbed device fires an interrupt on itself. Two Node.js threads are run, so that they do not interfere with one another:

1. *tx.js*: this process toggles a GPIO digital state, which is connected to another GPIO of the RPi, firing the interrupt; it stores the timestamp of the toggling process as well in microseconds;
2. *rx.js*: this process reacts to both rising and falling edge interrupts on the GPIO and stores the timestamp of the event in microseconds.

With this, we get two arrays of *equally referenced* timestamps: one corresponding to interruption command timestamps, the other corresponding to interruption reaction timestamps. The difference between them is the delay in charge of the Testbed device reacting to owned events. We set the *tx.js* script to fire an interrupt every 10 ms—100 per second. We let the test run for 24 h, thus firing more than eight million interruption events. 

**Figure 6:**
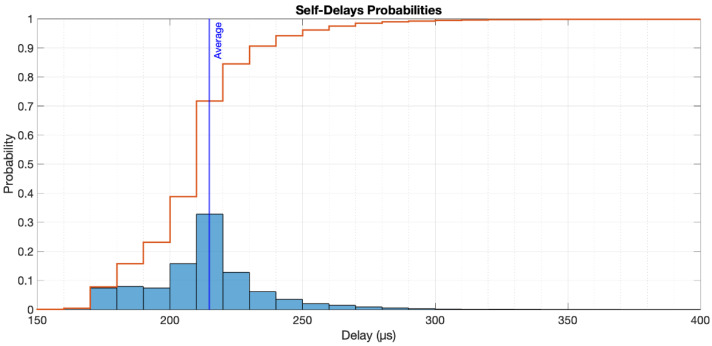
Self-delays probabilities.

The average delay was 214.85 µs, with a standard deviation of 96.99 µs. The delays are depicted in Figure 6, along with the corresponding cumulative distribution function. As can be seen, 90% of the time, the introduced delay for self-reacting events is <240 µs.

### 3.4. Testbed’s Error Range Validation

With the characterisation made in Section 2.2 and Section 2.3, we have all the temporal variabilities of our device, with which we can estimate its error margin:

- External interrupts: the response to external interrupts seems to be very accurate and precise, with 90 % of drifting within 12 µs. Since we are working with latencies on the order of tens of milliseconds, we can consider this influence negligible (~0.1%).
- Internal clock: the internal clock presents an error in the order of hundreds of microseconds (240 µs), with 90% confidence values between 160 µs and 370 µs.
- Self-delay: the self-delay, which is probably the most important factor in our system since we are using the same Testbed to command external devices and measure time, has an average value around 215 µs, with a 90% confidence delay <240 µs and no values less than 160 µs.

With these matters, one can realise that the influences are always cumulative, meaning that the latencies measured by our Testbed will always be longer than actual latencies. Considering this, we can claim that the real latency of a message will be as in Equation (1):

(1) γ=φ−δ,  δ∈[320, 610] μs 

where:

- *γ* is the actual latency;
- *φ* is the measured latency;
- *δ* is the introduced metering error, which is, according to our characterisation, a value in the range of 320 µs and 610 µs.

Thus, we can focus the result a little more, as in Equation (2):

(2) $$\gamma \;\left(\mathrm{ms}\right)=\varphi \;\left(\mathrm{ms}\right)-\delta^{\prime }-0.32,\;\;\delta ’\in \left[0,\;0.29\right]\;$$

**Figure 7:**
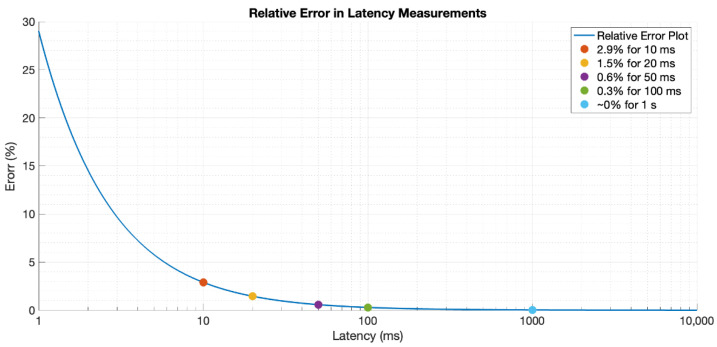
Testbed’s relative error with respect to the latency value.

Hence, the error of our system can be said to be less than 290 µs, which means a relative error around 3% for latencies on the order of tens of milliseconds, i.e., that of most IoT wireless technologies. This error gets reduced as the wireless IoT latency increases, being around 1.5% for 20 ms, 0.6% for 50 ms, 0.3% for 100 ms, and negligible from 1 s and on—see Figure 7.

## 4. Measurements and Results

In this Section, we show example results for the measurements our Testbed can perform, proving its ability to perform communication reliability measurements with no regard to the wireless technology under test or the hardware platform used as IoT device. In Section 4.1, the Testbed’s measuring parameters are presented; in Section 4.2 we analyse the performance of the following technologies, with expected latency in brackets [12]: 6LoWPAN (~20 ms), LoRaWAN (~300 ms), Sigfox (~4 s), Zigbee (~40 ms), Wi-Fi (~30 ms), BLE (~30 ms) and NB-IoT (~2 s); following is a discussion about the results obtained in Section 4.3.

### 4.1. Parameters: Latency, Error, Stability

**Figure 8:**
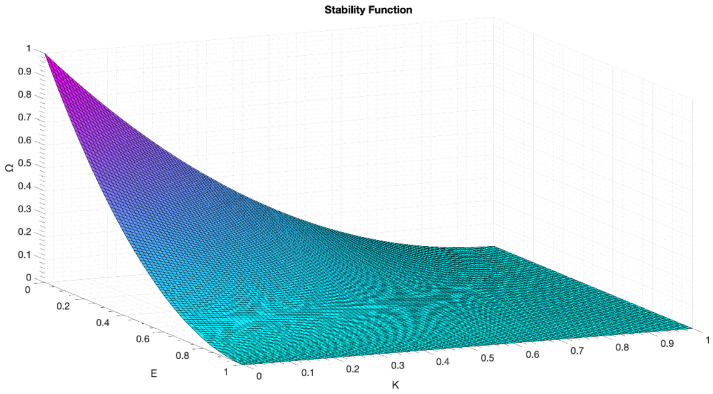
3D plot of the stability function.

The three parameters that we evaluate in this work are: latency, error rate and stability. The following data is presented for each matter:

- Latency: minimum, average, maximum;
- Error rate: message loss count (Γ), relative error rate (E);
- Stability: standard deviation of latency (Λ), messages out of average latency ± 10%—outliers, both in absolute count (Π) and relative (K)—and a factor indicating a quality of stability (Ω) defined as in Equation (3) and Figure 8.

(3) $$\Omega ={\left[\left(1-E\right)\times \left(1-K\right)\right]}^{2},\;\;\Omega ,E,K\in \left[0,\;1\right]\;$$

The stability function (Ω) serves as a rapid measurement of how accountable, reliable and congruent a designated technology in its performance is. It accounts for the variability of the latency and the number of losses. As can be seen, it rapidly decreases with any of the factors involved, as any small variation in the expected behaviour of the wireless protocol could eventually lead to fatal phenomena in critical applications—provided that strong temporal stability or message integrity is needed [32,33,34,35].

### 4.2. Real Tests

We performed tests for every wireless technology under characterisation to confirm the versatility of the Testbed device. In Table 4, we show the summary of results for tests with a period of transmissions (PT, seconds) and a message limit (ML, number) as follows:

- 6LoWPAN:    ML 10,000;    PT 1
- LoRaWAN:    ML 10,000;    PT 1
- Sigfox:      ML 100;      PT 15
- Zigbee:        ML 10,000;    PT 1
- Wi-Fi:       ML 10,000;    PT 1
- BLE:         ML 500;      PT 10
- NB-IoT:      ML 5000;     PT 5

These results function as a reference for every wireless technology in the scope, and the number of messages is representative so as to determine losses and stability—not for Sigfox due to its own limitations, but the results were consistent every time we repeated the test. It also confirms the universal aim of the Testbed developed. 

In Appendix B, the reader can see cropped plots for the results depicted in Table 4 as a visual reference of the latency behaviour. In these plots, one thousand messages are usually depicted—except for Sigfox (100) and BLE (500)—with a light blue area indicating the non-outlying zone for stability calculation and an orange horizontal line representing the average *latency*.

### 4.3. Results Discussion

The results obtained within this work comply with those related in the literature and the protocol spec sheets: tens of milliseconds for 6LoWPAN, Zigbee, Wi-Fi and BLE; hundreds of milliseconds for LoRaWAN; some seconds for Sigfox and NB-IoT. 

NB-IoT is the rarest scenario, since it is supposed to be a managed network with a strong infrastructure, and even quality of service (QoS). However, it presents a hard oscillatory behaviour with strong latency peaks, some of which even reach more than ten seconds—although this fact complies with the 3GPP Release-13 target of ≤10 s latency for 99% of messages [36].

Furthermore, we must consider that specifically in the case of NB-IoT, data travels through a backend of which we do not have any influence or knowledge. This backend (Pycom Pybytes) could be disturbing measurements and therefore be responsible for the incongruent, appealing peaks. The IoT device used as a transmitter device could also be misbehaving and altering the results. Nonetheless, this does not seem much of an option as this behaviour did not happen with Sigfox, which also relies on a public network and proprietary backend to work and for which we used the same development board as transmitter device (Pycom FiPy).

The selected devices always play some role in the final observed latencies as well as the specific topology actually deployed and even the software implemented. However, we can claim that the Testbed achieved its universal purpose, as we can measure latencies for a wide variety of wireless technologies and hardware platforms with truthful, consistent results. Furthermore, we demonstrated a proper accuracy for the Testbed with an error in the range of just 0–290 µs.

The selection of one technology over another would depend on the application requirements. On the one hand, thanks to the stability definition, we can have an overview of the uniformity in latency and error rate. On the other hand, with the latency measurement we get to know the immediacy in response to an event.

Generally, if the data are too sensitive (error and stability) and immediacy (latency) is not a strong requirement, one may choose Sigfox or LoRaWAN. If more immediacy is required, Zigbee and 6LoWPAN happen to be the perfect choice. Provided we need a high immediacy but stability is not a crucial factor, Wi-Fi tends to be an option. 

In spite of that, if the use case had other specific key requirements, superseding latency, error rate and stability, those must be the decisive ones. For instance, if cost and low energy consumption play a key role, BLE would be the choice, but NB-IoT would be the preferred option for applications deployed in a wide area with hundreds or thousands of sensors.

It is noticeable that Zigbee, Wi-Fi and BLE present no losses. We point this to the following facts:

- Zigbee: we used the whole Zigbee stack, which handles losses itself.
- Wi-Fi: we used HTTP, which works over Transmission Control Protocol (TCP) and therefore handles losses as well.
- BLE: as stated in Section 2.2 hereof, we advertised every message twice, so the chance for the message getting lost is negligible.

Yet, it was no surprise that Sigfox and NB-IoT provided no losses. In the case of Sigfox it is due to the robustness of Sigfox’s ultra-narrow band (UNB) modulation and the fact that every message is sent three times in three different carriers [37,38]—with the counterpart of slowness. In the case of NB-IoT, it uses a proprietary band with restricted access and QoS, and NB-IoT has its target in handling more than 50,000 devices per sector [36], which is very far from actuality at the moment of doing this work.

## 5. Conclusions

In this work, we presented a universal Testbed to characterise IoT wireless technologies in three crucial factors: latency, error rate and stability. The Testbed was characterised in Section 3, where we demonstrated its ability to measure time-sensitive matters—such as latency—with an error of 3%: <290 µs for typical IoT latencies around tens of milliseconds. For the wireless IoT technologies characterised in this work as exemplification, the results obtained fell in the expected range, which also helps validate the Testbed’s performance.

The main advantages of this Testbed device are:

- Technology-agnostic: it has been designed to characterise any IoT wireless technology, with independence of the communication architecture and the protocols used;
- Cost, time, and resource efficient: it is based on the affordable Raspberry Pi board, and it can work on any Linux machine with a Node.js runtime;
- Portable and easy to deploy: the Testbed design allows its transportation and set up in any location;
- Replicable and scalable: based on standard HW and SW tools, it is easy to replicate, adapt and improve to measure other timing parameters.

### Future Sights

To enhance the work in this matter, more technologies should be evaluated to keep the universal track of the Testbed device, such as Thread—based on 6LoWPAN and part of the future Matter [39] or GPRS—which are still widely used nowadays in machine-to-machine (M2M) communications.

**Figure 9:**
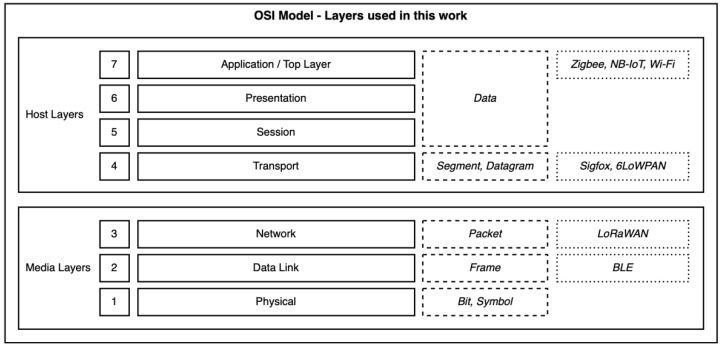
OSI model layer reference related to the specific layers we used with each wireless technology, showing its primary function, and data meaning and contents.

Furthermore, the different IoT protocols should be more equated in terms of open systems interconnection (OSI) layers—although the OSI model (see Figure 9) may not be truly accurate for all of these IoT wireless technologies and some terminology might change, it serves as a reference [40,41,42,43,44,45,46]. In the tests performed within this work, we evaluated the following technologies at different layers:

- Applications: Zigbee, NB-IoT, Wi-Fi (HTTP);
- Transport: Sigfox, 6LoWPAN (UDP);
- Network: LoRaWAN;
- Data Link: BLE (advertisements, two repetitions per message).

As one can see, different layers were used in different technologies, and some of them already implement recovery mechanisms or error mitigations. Moreover, there are limitations regarding some technologies in the use of certain layers:

- Zigbee, Sigfox, LoRaWAN: due to their own nature and definition, those layers are the ones to use between the end device and the receiver on the RF link;
- BLE: the equipment used in this work (Pycom FiPy), only supported BLE advertisements when doing these tests—other manufacturers may provide support for upper layers;
- NB-IoT: at the moment of this work, we could only use Pybytes’ abstraction layer to send and receive NB-IoT messages; so, we are considering this the top layer.

Nonetheless, using the aforementioned layers is significative for IoT wireless technologies characterisation, as they are those of the most used layers in each technology. Yet, for 6LoWPAN, we could implement constrained application protocol (CoAP) or MQTT on the application layer to perform these tests as well because they are the most used top-layer-protocols for this technology. Into the bargain, even high-level, novel, IoT-targeted enhancements for future-proving existing wireless technologies could be tested, such as that in [47], in which Chen et al. present a Wi-Fi modification specifically designed to accommodate the large amount of data and nodes the IoT brings, or that in [48], in which Magsi et al. propose an adaptive data transmission framework for healthcare applications.

Additionally, it could be useful to implement a graphical web interface to display results and command tests apart from the command-line interface we have so far, and implementing some sort of logarithmic function—or other function of interest—to the period between messages could be helpful to perform stress tests, as the period would shrink continuously until an error threshold was reached.

## Figures and Tables

**Table 1:** Physical equipment, depending on the wireless technology.

Wireless Technology	RF Link Band	Transmitter	Receiver	Gateway/Internet
6LoWPAN	ISM 2.4 GHz	T.I. CC2538 ARM Based Mote	T.I. CC2538 ARM Based Mote	*gateway:* BeagleBone Black
LoRaWAN	ISM 868 MHz	Pycom FiPy	Semtech SX1301 Based Mote	*gateway:* Raspberry Pi 4
Sigfox	ISM 868 MHz	Pycom FiPy	Sigfox Backend	*public Internet needed*
Zigbee	ISM 2.4 GHz	SiLabs MGM210P	SiLabs MGM210P	*gateway:* SiLabs SLWSTK6102A
Wi-Fi	ISM 2.4 GHz	Pycom FiPy	Raspberry Pi 4	*none*
BLE	ISM 2.4 GHz	Pycom FiPy	Raspberry Pi 4	*none*
NB-IoT	LTE band 20	Pycom FiPy	Vodafone Network	*public Internet needed*

**Table 2:** Observed periods to external interrupts: important values.

Deterministic Low Width	Observed Avg. Low Period	Deterministic High Width	Observed Avg. High Period	90% Confidence ∆T
9.9555 ms	9.9556 ms	10.0448 µs	10.045 ms	12 µs

**Table 3:** Temporal stability of the Testbed’s internal clock.

Target	Measured	Error	Relative Error	–10 dB BW ^1^
50.0 Hz 20.0 ms	49.4 Hz 20.24 ms	0.6 Hz 0.24 ms	1.2%	0.5 Hz 49.1–49.6 Hz

^1^ Range of frequency around the central peak for which the magnitude of the FFT is above a 10% of the peak magnitude.

**Table 4:** Results summary.

Measurement		6LoWPAN	LoRaWAN	Sigfox	Zigbee	Wi-Fi	BLE	NB-IoT
Latency (ms)	Min.	19.522	282.40	3467.1	34.174	25.294	13.382	329.29
	Avg.	22.116	296.96	3695.2	48.298	32.300	26.974	1797.3
	Max.	356.14	334.81	5651.0	95.295	178.10	125.40	10,275
Error	Γ	2	66	0	0	0	0	0
	E	0.02%	0.66%	0%	0%	0%	0%	0%
Stability	Λ (ms)	9.883	5.419	290.4	5.242	9.502	13.68	1352
	Π	386	2	4	2307	3197	480	4052
	K	3.86%	0.02%	4%	23.1%	31.9%	96.0%	81.0%
	Ω	0.924	0.993	0.922	0.592	0.463	0.002	0.036

**Figure A2:**
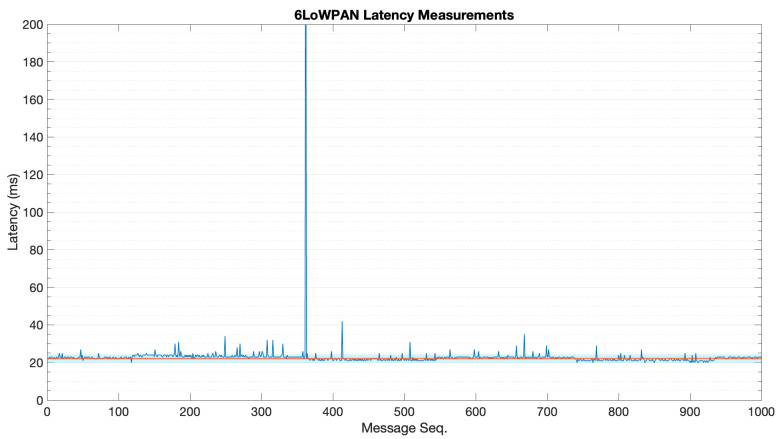
Latency plot for 6LoWPAN.

**Figure A3:**
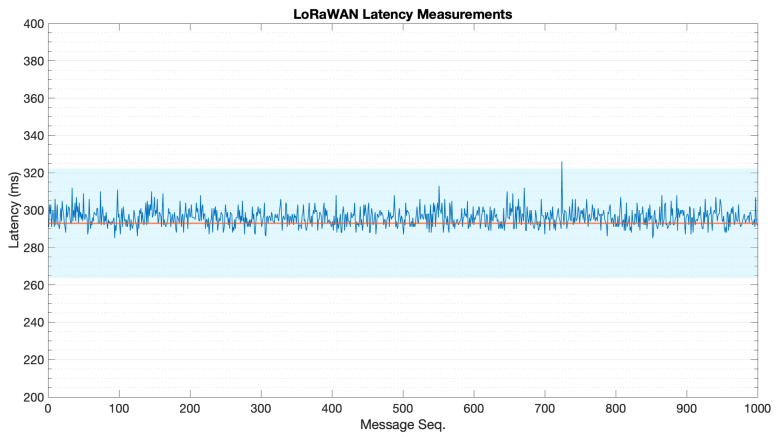
Latency plot for LoRaWAN.

**Figure A4:**
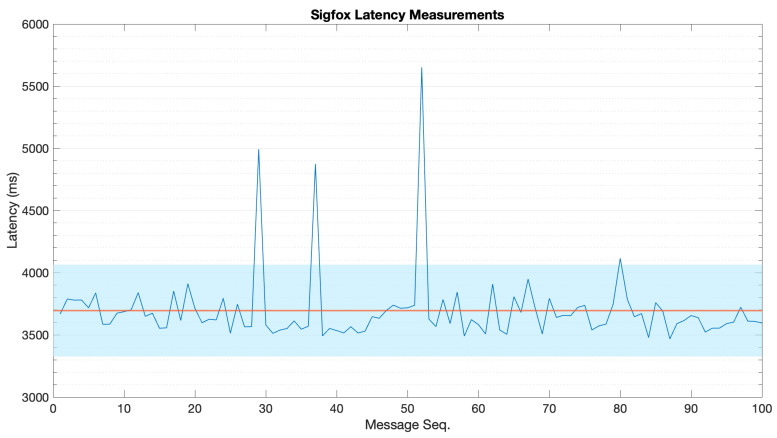
Latency plot for Sigfox.

**Figure A5:**
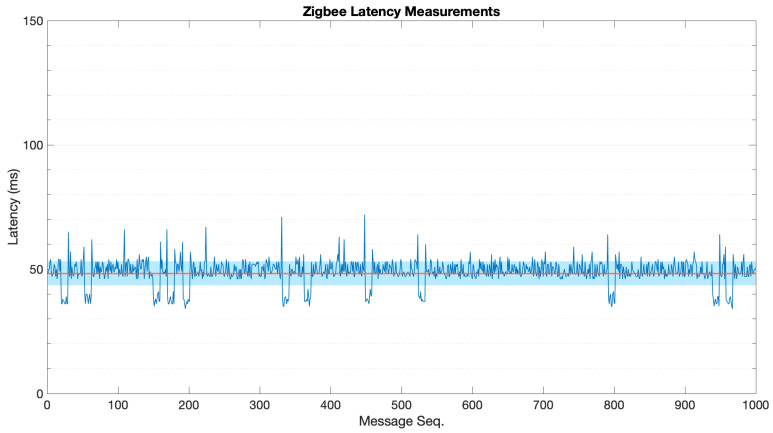
Latency plot for Zibgee.

**Figure A6:**
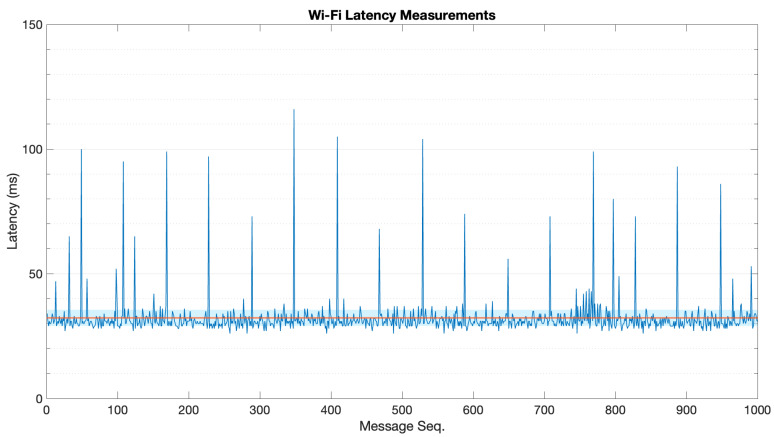
Latency plot for Wi-Fi.

**Figure A7:**
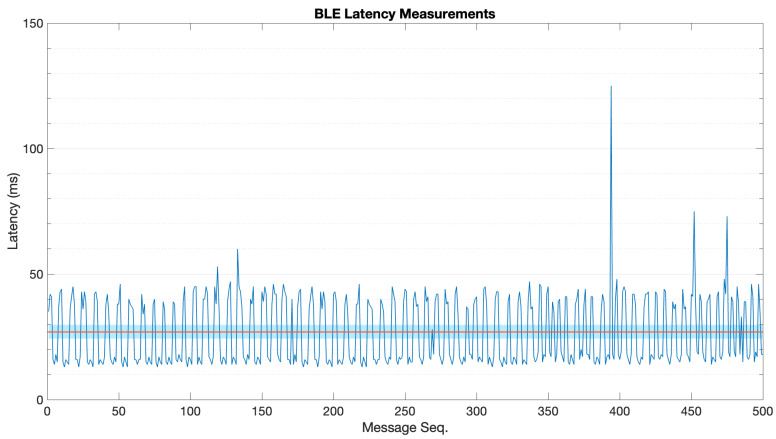
Latency plot for BLE.

**Figure A8:**
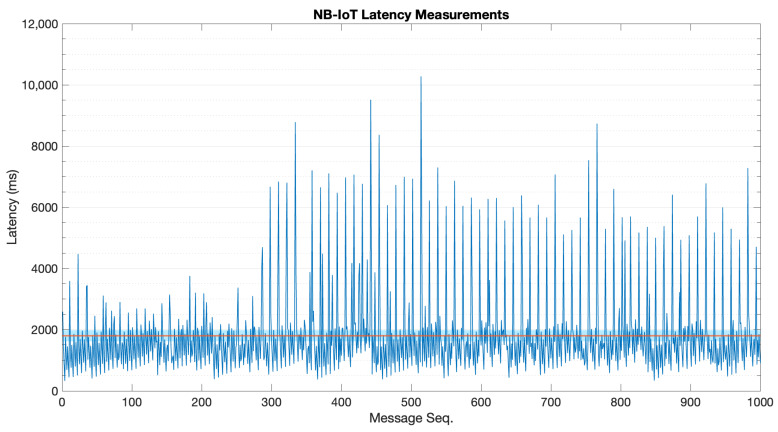
Latency plot for NB-IoT.

## Data Availability

Not applicable.

## Appendix A

This appendix contains an additional diagram for Section 2—Figure 1.

## Appendix B

This appendix contains plots for the latency measurements presented in Table 4.
